# Effects of brimonidine tartrate 0.1% ophthalmic solution on the pupil, refraction, and light reflex

**DOI:** 10.1038/s41598-018-27436-8

**Published:** 2018-06-13

**Authors:** C. O. Sayaka Kato, Kimiya Shimizu, Kazutaka Kamiya, Hitoshi Ishikawa, Akihito Igarashi

**Affiliations:** 1 Eye Center, Sanno Hospital, Tokyo, Japan; 2 Department of Ophthalmology, University of Kitasato School of Medicine, Kanagawa, Japan

## Abstract

This study aimed to assess changes in pupil size, uncorrected visual acuity, refraction, and the direct and consensual light reflexes after instillation of brimonidine 0.1% in healthy subjects. The investigation comprised 46 eyes of 23 healthy subjects with no eye diseases in whom brimonidine 0.1% was instilled in the right eye. Pupil size was measured quantitatively under photopic and scotopic conditions, uncorrected visual acuity, refraction, and direct and consensual light reflexes before and at 1, 6, and 24 h after instillation. We found No significant change was found in refraction or uncorrected visual acuity (*P* = 0.999 and *P* = 0.998, respectively). A significant reduction in pupil size was observed under scotopic conditions at 1 h and 6 h after instillation (*P* = 0.007 and *P* = 0.005, respectively). The rate of pupil contraction and constriction speed measured by light reflexes were significantly increased at 1 h and 6 h after instillation (*P* = 0.021 and *P* = 0.033, respectively). Brimonidine 0.1% induced a significant reduction in pupil size under scotopic conditions without a significant change in refraction or visual acuity due to suppression of the sympathetic nervous system.

## Introduction

After refractive surgery, patients may experience problems with nighttime glare and halos^1^. Currently, concerns exist regarding these issues, which deteriorate visual performance and subsequent patient satisfaction. A reduction in night vision occurs due to increases in the high-order aberrations that occur with large pupils; however, this may be improved by miosis^2,3^. Nighttime glare and halos have been reported to improve with miosis from pilocarpine hydrochloride. However, pilocarpine has been reported to cause excessive miosis, increased lens thickness, and adjustable tension from increases in transient myopia^4^.

Brimonidine, a selective alpha-2 adrenergic receptor agonist, is used in the treatment of glaucoma and ocular hypertension. Its reduces intraocular pressure by reducing aqueous humor production and increasing uveoscleral outflow^5,6^. Brimonidine 0.2% peak ocular hypotensive effect occurs 2 h after instillation^7^, and there was no significant difference from timolol maleate at the maximum effect^8^. Therefore, it can be expected to provide effective intraocular pressure reduction in combination with other ophthalmic solutions. Brimonidine has been reported to have neuroprotective effect^9,10^. Krupin *et al*.^11^ reported that low-pressure glaucoma patients treated with brimonidine 0.2% are less likely to experience field progression. Brimonidine tartrate 0.2% and 0.15% ophthalmic solution has been reported to be significantly miotic under scotopic conditions^12,13^, and effective in reducing glare and halos after refractive surgery^14,15^. The pupil size contracts under scotopic conditions is that brimonidine stimulates prejunctional alpha-2 agonist receptor and reduces norepinephrine release in the synapse. Therefore, brimonidine inhibits pupil dilation under scotopic vision. Although previous studies have investigated pupil size, there have been no reports investigating a detailed description of direct and consensual light reflexes. The aim of this study was to assess changes in pupil size, uncorrected visual acuity, corrected visual acuity, subjective refraction, objective refraction, and the direct and consensual light reflexes after instillation of brimonidine 0.1% in the eyes of healthy subjects.

## Results

**Figure 1:**
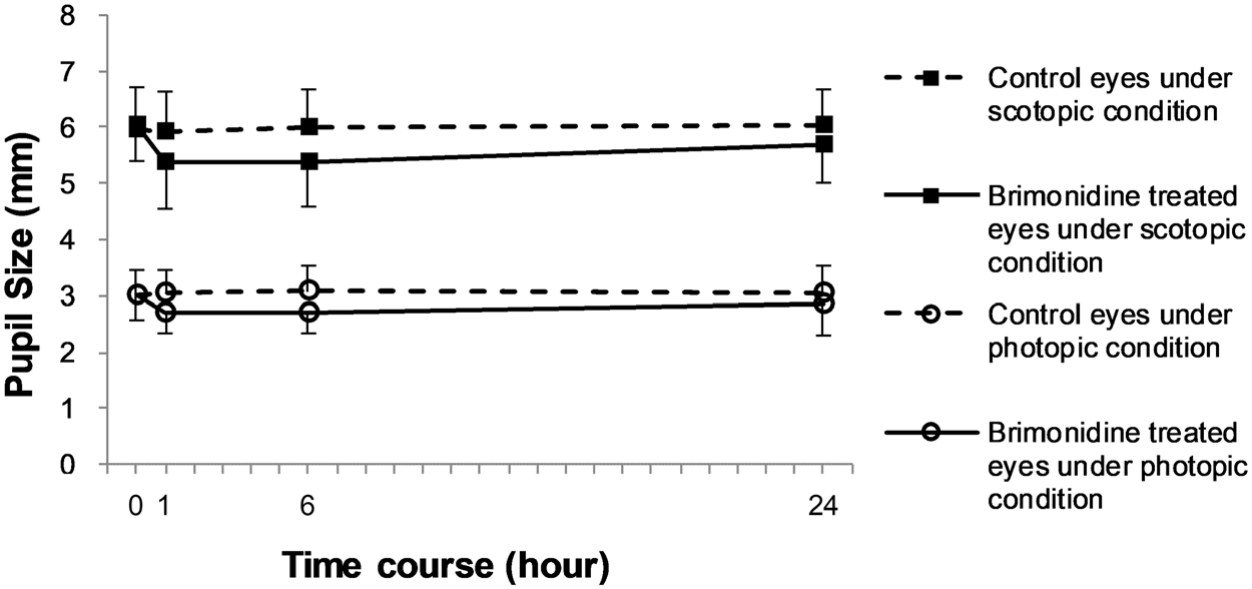
Pupil size in brimonidine-treated eyes and control eyes under photopic and scotopic conditions.

All of the results were examined at 1, 6, and 24 h after instillation compared with pre-instillation. We found no significant change in pupil size in the brimonidine-treated eyes under photopic conditions, but a significant reduction in pupil size under scotopic conditions at 1 h (5.4 ± 0.8 mm) and 6 h (5.4 ± 0.8 mm) after instillation (*P* = 0.007 and *P* = 0.005, respectively) compared to before instillation of brimonidine (6.0 ± 0.6 mm) (Fig. 1, Table 1). We found no significant change in pupil size in the brimonidine-treated eyes under scotopic conditions at 24 h (5.7 ± 0.7 mm) after instillation (*P* = 0.144). The pupil size in the control eyes was not significantly changed under photopic or scotopic conditions (Fig. 1). We found no significant change in uncorrected visual acuity, subjective refraction, or objective refraction (Table 2). The logarithm of the minimal angle of resolution (logMAR) of corrected visual acuity was −0.18 in all cases before and after instillation.

**Table 1:** Pupil size in brimonidine-treated eyes before, and at 1, 6, and 24 h after instillation.

Pupil size	Pre	1 h	6 h	24 h	*P*-value
Under photopic conditions (mm)	3.0 ± 0.5	2.7 ± 0.4	2.7 ± 0.4	2.9 ± 0.6	0.067
Under scotopic conditions (mm)	6.0 ± 0.6	5.4 ± 0.8**	5.4 ± 0.8**	5.7 ± 0.7	0.011

There was no significant change in pupil size in the brimonidine-treated eyes under photopic conditions after instillation. The pupil size under scotopic conditions was significantly reduced at 1 h and 6 h after instillation (*P* = 0.007 and *P* = 0.005, respectively). There was no significant change in pupil size under scotopic conditions at 24 h after instillation (*P* = 0.144).

*P < 0.05; **P < 0.01; ***P < 0.001. Pre = before instillation of brimonidine.

**Table 2:** Uncorrected visual acuity and refraction on brimonidine treated eyes before, and at 1, 6, and 24 h after instillation.

Variable	Pre	1 h	6 h	24 h	*P*-value
Uncorrected visual acuity (logMAR)	0.71 ± 0.61	0.65 ± 0.60	0.65 ± 0.60	0.69 ± 0.60	0.998
Subjective refraction (D)	−2.97 ± 2.77	−2.75 ± 2.78	−2.56 ± 2.77	−2.64 ± 2.76	0.999
Objective refraction (D)	−3.72 ± 2.94	−3.76 ± 2.95	−3.59 ± 3.06	−3.68 ± 3.02	0.998

Data presented as mean ± SD, unless otherwise indicated. There was no change in uncorrected visual acuity, subjective refraction, and objective refraction after instillation.

logMAR = logarithm of the minimal angle of resolution; D = Diopter; Pre = before instillation of brimonidine.

Regarding the results of the direct light reflex in the brimonidine-treated eyes, initial pupil size was significantly reduced at 1 h (5.3 ± 0.9 mm) and 6 h (5.4 ± 0.9 mm) after instillation (*P* = 0.001 and *P* = 0.002, respectively) compared to before instillation of brimonidine (6.3 ± 0.7 mm) (Table 3). Minimum pupil size after light stimulation was also significantly reduced at 1 h (3.3 ± 0.7 mm) and 6 h (3.4 ± 0.7 mm) after instillation (*P* < 0.001 and *P* < 0.001, respectively) compared to before instillation of brimonidine (4.2 ± 0.7 mm). Rate of pupil contraction [(Initial pupil size − minimum pupil size after light stimulation)/(initial pupil size)] was significantly increased at 1 h (0.37 ± 0.06) and 6 h (0.37 ± 0.04) after instillation (*P* = 0.020 and *P* = 0.037, respectively) compared to before instillation of brimonidine (0.33 ± 0.07). Time until the pupil size was minimized was significantly reduced at 1 h (863.8 ± 169.8 ms) and 6 h (871.7 ± 200.5 ms) after instillation (*P* = 0.010 and *P* = 0.015, respectively) compared to before instillation of brimonidine (1018.8 ± 141.4 ms). Time when the pupil size had been restored to 63% was not observed to change significantly at any time point. Maximum constriction speed was significantly increased at 1 h (5.6 ± 0.9 mm^2^/s) and 6 h (5.7 ± 0.8 mm^2^/s) after instillation (*P* = 0.027 and *P* = 0.016, respectively) compared to before instillation of brimonidine (5.0 ± 0.8 mm^2^/s). Maximum dilatation speed did not change significantly at any time point. No significant change in any parameter from baseline was observed at 24 h after instillation. The results of the indirect reaction in the brimonidine-treated eyes were similar to the results of the direct reaction (Table 4). No parameter was observed to change significantly in the control eyes at any time point. In addition, no side effects were observed in any case.

**Table 3:** The direct light reflex on brimonidine-treated eyes before, and at 1, 6, and 24 h after instillation.

Variable	Pre	1 h	6 h	24 h	*P*-value
Initial pupil size (mm)	6.3 ± 0.7	5.3 ± 0.9**	5.4 ± 0.9**	5.8 ± 0.9	0.001
Minimum pupil size after light stimulation (mm)	4.2 ± 0.7	3.3 ± 0.7***	3.4 ± 0.7***	3.8 ± 0.7	<0.001
Rate of pupil contraction****	0.33 ± 0.07	0.37 ± 0.06*	0.37 ± 0.04*	0.34 ± 0.05	0.021
Time until the pupil size was minimized (ms)	1018.8 ± 141.4	863.8 ± 169.8*	871.7 ± 200.5*	918.9 ± 182.0	0.013
Time when the pupil size had been restored to 63% (ms)	1472.5 ± 353.6	1591.3 ± 318.4	1502.9 ± 264.5	1531.9 ± 406.1	0.676
Maximum constriction speed (mm^2^/s)	5.0 ± 0.8	5.6 ± 0.9*	5.7 ± 0.8*	5.2 ± 0.8	0.033
Maximum dilatation speed (mm^2^/s)	2.3 ± 0.4	2.1 ± 0.4	2.2 ± 0.4	2.2 ± 0.4	0.784

The direct light reflex in the brimonidine treated eyes, initial pupil size was significantly reduced at 1 h and 6 h after instillation (*P* = 0.001 and *P* = 0.002, respectively). The rate of pupil contraction was significantly increased at 1 h and 6 h after instillation (*P* = 0.020 and *P* = 0.037, respectively) *P < 0.05; **P < 0.01; ***P < 0.001. Pre = before instillation of brimonidine

****(Initial pupil size - minimum pupil size after light stimulation)/(initial pupil size).

**Table 4:** The consensual light reflex on brimonidine-treated eyes before, and at 1, 6, and 24 h after instillation.

Variable	Pre	1 h	6 h	24 h	*P*-value
Initial pupil size (mm)	6.2 ± 0.8	5.3 ± 0.9**	5.3 ± 1.1**	5.8 ± 0.9	0.002
Minimum pupil size after light stimulation (mm)	4.2 ± 0.7	3.3 ± 0.7***	3.3 ± 0.8***	3.8 ± 0.7	<0.001
Rate of pupil contraction****	0.33 ± 0.06	0.38 ± 0.05**	0.38 ± 0.05**	0.35 ± 0.05	0.003
Time until the pupil size was minimized (ms)	1023.2 ± 129.3	894.9 ± 162.5*	836.8 ± 266.2**	986.2 ± 155.3	0.004
Time when the pupil size had been restored to 63% (ms)	1434.8 ± 326.7	1580.4 ± 284.6	1553.6 ± 268.8	1497.1 ± 279.4	0.339
Maximum constriction speed (mm^2^/s)	4.9 ± 0.9	5.7 ± 0.8**	5.7 ± 0.9**	5.3 ± 0.6	0.002
Maximum dilatation speed (mm^2^/s)	2.2 ± 0.4	2.0 ± 0.3	2.2 ± 0.3	2.3 ± 0.5	0.399

The results of the indirect reaction in the brimonidine-treated eyes were similar to the results of the direct reaction.

*P < 0.05; **P < 0.01; ***P < 0.001. Pre = before instillation of brimonidine.

**** (Initial pupil size - minimum pupil size after light stimulation)/(initial pupil size).

## Discussion

We found that pupil size was significantly reduced under scotopic conditions at 1 h and 6 h after instillation (0.64 mm and 0.67 mm, respectively). The rate of pupil contraction and maximum constriction speed were significantly increased at 1 h and 6 h after instillation. McDonald *et al*.^12^ found no significant change in pupil size under photopic conditions; however, 100% (16 eyes) exhibited significant miosis after 30 min, and 81.3% (13 eyes) miosis at 6 h under scotopic conditions after brimonidine 0.2% instillation in healthy subjects. The results reported by McDonald *et al*. did not differ from those in the present study in that pupil size before instillation under photopic conditions was 2.9 mm, and 2.8 mm 6 h after instillation. However, pupil size under scotopic conditions was 5.8 mm before instillation and 4.4 mm 6 h after instillation, which is miotic compared with our study. One of possibility is that the study by McDonald *et al*. used a 0.2% concentration of eye drops compared with 0.1% in our study. Kesler *et al*.^16^ found significant miosis after brimonidine 0.2% instillation in healthy subjects up to 4 h under photopic conditions and up to 6 h under scotopic conditions. The authors reported a pupil size of 4.81 mm before instillation under photopic conditions and 3.80 mm 4 h after instillation, which is significantly miotic. Because the photopic condition is defined at 5 cd/m^2^, we believe that the pupil size was larger than that in our study, and significantly miotic. Shemesh *et al*.^13^ found no significant change in pupil size under photopic conditions; however, a significant reduction in pupil size under scotopic conditions up to 6 h after brimonidine 0.1% instillation was found in healthy subjects. Because Shemesh *et al*. used 5 cd/m^2^ under photopic conditions, the pupil size before instillation was as large as 4.98 mm from this study. However, the pupil size 6 h after instillation was 4.64 mm, which is not considered to be a significant difference because 0.1% brimonidine was used. Therefore, pupil size 6 h after instillation under scotopic conditions was 5.38 mm, which is similar to the result in the present study. Thordsen *et al*.^17^ reported measurements of pupil size after 0.15% brimonidine was instilled in healthy subjects. Under scotopic conditions, pupil size was decreased by ≥1.0 mm in 100% and 60% of eyes at 30 min and 6 h, respectively. The instruments used to measure pupil size in previous studies were types of closed-view pupilometers. In the present study, measurements were performed with both eyes open because this is considered to provide a more accurate measurement of pupil size. In addition, the past study reported measurements up to 6 h after instillation. In our study, it was measured for 24 h after instillation, with no significant difference from 24 h before instillation found.

Direct and consensual light reflexes under scotopic conditions were compared with reports of pupil diameter under scotopic conditions because of miosis 6 h after instillation and were considered to be similar. Parameters of light reflexes are related to miosis and those related to mydriasis. Parameters related to miosis include initial pupil size, minimum pupil size after light stimulation, rate of pupil contraction, time until pupil size is minimized, and maximum constriction speed. Parameters related to mydriasis include time to pupil size restored to 63%, and maximum dilatation speed. Parameters related to miosis in pupils significantly miotic at 1 and 6 h after instillation include increased rates of pupil contraction and maximum constriction speed. In contrast, no significant difference was observed in the two parameters related to mydriasis after instillation, and brimonidine did not affect mydriasis. In addition, this result is the same for consensual light reflexes, and it is understood that brimonidine also similarly acts through an indirect reaction route.

There were at least two limitations to this study, the first of which was the relatively small sample size. A larger number of healthy subjects is required to confirm our preliminary findings. Second, this study was performed in an unblinded fashion. A double-blinded study may be more appropriate to confirm the veracity of the results.

Pilocarpine hydrochloride, a parasympathetic stimulant, has been reported to increase myopia and the perception of darkness due to excessive miosis; it is considered unsuitable to reduce night vision glare and halos after refractive surgery. Pupil size was significantly reduced under scotopic conditions at 1 h after instillation of brimonidine. When used for nighttime glare and halos, we believe that it is effective once per day when instilled in the evening. Brimonidine has slight miotic properties under scotopic conditions by suppressing the sympathetic nervous system. There was no significant change in pupil size under photopic conditions; therefore, the pupil was also considered to be a factor that did not significantly influence uncorrected visual acuity. Brimonidine 0.1% induced a significant reduction in pupil size without a significant change in refraction or visual acuity under scotopic conditions. Therefore, it may be effective for the reduction of glare and halos after refractive surgery.

## Materials and Methods

This study comprised 46 eyes of 23 healthy subjects (13 men, 10 women; mean age 31.4 ± 7.3 years) with no eye diseases other than refractive error. Brimonidine 0.1% (Alphagan®, Senju Pharmaceutical Co., Ltd., Osaka, Japan) was instilled in the right eye and artificial tears were instilled in the left; we quantitatively measured pupil size under photopic and scotopic conditions, uncorrected visual acuity, corrected visual acuity, subjective refraction, objective refraction, and the direct and consensual light reflexes before and at 1, 6, and 24 h after instillation. In all cases, the time measurements were unified: measurements were made at 8:30 AM, instillation was performed at 9:00 AM, and outcomes were measured at 1, 6, and 24 h after instillation. The subjects were instructed to refrain from smoking and ingesting caffeine, since these can affect the pupil. Pupil size was measured with both eyes open using an electronic pupil meter (FP-10000 II; T.M.I. Company, Saitama, Japan) and was quantitatively measured under photopic and scotopic conditions (130 cd/m^2^ and 0.1 cd/m^2^, respectively). Objective refraction was measured using a combination system including an auto refractometer, auto keratometer, non-contact tonometer, and non-contact pachymeter (Auto Ref Kerato Meter; TONOREF II^®^; NIDEK CO., LTD., Aichi, Japan). The direct and consensual light reflexes was measured with an infrared iriscorder (Iriscorder Dual C-10641, Hamamatsu Photonics Co., Ltd., Shizuoka, Japan); it measured the direct and indirect reactions of both eyes to red light stimulation (635 nm) after scotopic adaptation for 15 min.

The direct and consensual light reflexes parameters were initial pupil size, minimum pupil size after light stimulation, rate of pupil contraction, time until the pupil size was minimized, time when the pupil size had been restored to 63%, maximum constriction speed, and maximum dilatation speed. The instilled right eyes were defined as the brimonidine-treated eyes and the left eyes were defined as the control eyes. The study was approved by the Institutional Review Board at Kitasato University School of Medicine, and followed the tenets of the Declaration of Helsinki. Informed consent was obtained from all subjects. All statistical analyses were performed off-line using spreadsheet software (Excel, Microsoft Corporation, Redmond, WA, USA). Because all data were normally distributed, all statistical analysis was performed using the ANOVA test (Dunnett’s test); p < 0.05 was considered to be statistically significant.
